# A network meta-analysis of different acupuncture modalities in the treatment of bronchial asthma

**DOI:** 10.1186/s12890-023-02645-8

**Published:** 2023-09-22

**Authors:** Xingyi Wang, Shuyun Zeng, Zhuying Li, Yue Li, Hongtao Jia

**Affiliations:** 1 Heilongjiang university of Chinese Medicine, Harbin, Heilongjiang 150040 China; 2 First Affiliated Hospital, Heilongjiang University of Chinese Medicine, Harbin, Heilongjiang 150040 China

**Keywords:** Acupuncture, Bronchial asthma, Network meta-analysis, Randomized controlled trials

## Abstract

**Background:**

Glucocorticoids and Beta-2 receptor agonists are commonly used for the treatment of asthma in clinical practice, while these agents are accompanied by adverse reactions of different kinds. Studies have shown that acupuncture is effective in treating bronchial asthma. However, different acupuncture modalities have different costs and skill requirements, and there remains a lack of comparisons between different acupuncture modalities. This study aims to assess the efficacy of various acupuncture modalities in the treatment of asthma.

**Methods:**

The following databases were searched for randomized controlled trials (RCTs) on acupuncture for the treatment of bronchial asthma from database inception to August 26, 2022: PubMed, Embase, The Cochrane Library, Web of Science, Chinese Journal Full-text Database (CNKI), Wanfang Database (Wanfang Date), VIP Database (VIP), China Biology Medicine disc (CBM). Stata 15.1 software was used to conduct network meta-analysis. The risk of bias in the included studies was evaluated using the Cochrane Risk of Bias Assessment Tool 2 (RoB2).

**Results:**

A total of 8,693 relevant studies were found, and 30 RCTs were included, involving 2,722 patients with bronchial asthma and eight acupuncture modalities: manual acupuncture, moxibustion, electroacupuncture, ignipuncture, flying needle acupuncture, acupoint catgut embedding, acupoint application, and warm-needle moxibustion. The other 29 studies had certain risks, with the quality graded as “moderate”. Among the pair-wise comparisons of statistical significance (*p* < 0.05), acupoint application was the most effective in improving pulmonary function (FEV1: Traditional medicine therapy-acupoint application [-7.29 (-12.11,-2.47)]; acupoint application-moxibustion [7.20 (0.28,14.11)]; FVC: acupoint application-Traditional medicine therapy [8.02 (2.54,13.50)]). Acupoint catgut embedding was the most effective in improving the ACT score of the patients (Traditional medicine therapy-acupoint catgut embedding [-4.29 (-7.94, -0.65)]; acupoint catgut embedding-moxibustion [5.52 (1.05,9.99)]).

**Conclusion:**

Acupoint application has evident merits in improving the clinical response rate and pulmonary function, while acupoint catgut embedding can improve other secondary indicators. For the clinical treatment of asthma, acupoint application can be selected as a complementary and alternative therapy, while the other acupuncture therapies can also be considered according to the examination results of the patients.

**Supplementary Information:**

The online version contains supplementary material available at 10.1186/s12890-023-02645-8.

## Introduction

Asthma is a heterogeneous disorder characterized by chronic airway inflammation, with symptoms of wheezing, shortness of breath, chest tightness, cough, and varying degrees of exhalatory airflow restriction. These symptoms are often caused by factors such as exercise, allergen or irritant exposure, weather changes, or viral respiratory infections [1]. Glucocorticoids, β-2 receptor agonists, leukotriene modifiers, theophylline drugs, and anticholinergic drugs are often used for the clinical treatment of asthma, but all of them have different adverse effects [2]. Epidemiology shows that the number of asthma patients worldwide has reached 358 million [3]^.^ Repeated attacks of bronchial asthma seriously affect the quality of life of patients and bring heavy financial burdens. Therefore, it is urgent to find alternative therapies that are highly effective and have few adverse effects. Acupuncture has a broad range of indications [4], and is a crucial complementary and alternative therapy recommended by the World Health Organization (WHO) for treating asthma. It has multiple treatment modalities such as manual acupuncture, moxibustion, ignipuncture, electroacupuncture, warm-needle moxibustion, acupoint application, and acupoint catgut embedding.

Studies [5] have shown that acupuncture could alleviate airway remodeling and reduce airway resistance by regulating various signal pathways. It has a bidirectional regulative effect on multiple immune cells and molecules, and presents reliable therapeutic effects for inflammation, malignancies, infection, and autoimmune diseases [6]. A meta-analysis [7] has found that, compared with conventional treatments, acupuncture would be more effective in the treatment of asthma. However, due to the diversity of acupuncture modalities in clinical practice and their own advantages, it is easy to make clinicians confused when selecting an appropriate acupuncture therapy. At the same time, few clinical studies directly compared the efficacy of these therapies. Network meta-analysis is commonly used to assess the evidence network that involves more than 3 interventions, which includes direct and indirect comparisons and performs weighted and pooled analyses based on meta-analysis method. It processes sampling variation, heterogeneity of the interventions, and inconsistencies among the studies by constructing a hierarchical model and provides its maximum likelihood ratio [8]. This method can simultaneously compare the differences in therapeutic effects among multiple interventions in the evidence network and rank them according to the effect size, thus providing an important reference for clinical decision-making [9].

The purpose of this study was to investigate the efficacy of different acupuncture methods in the treatment of bronchial asthma. Since the patient compliance to the traditional medicine therapy is poor, there is an increased risk of disease progression [1]. This study helps in choosing an optimal acupuncture therapy as a substitute for the traditional medicine therapy, and provides a new idea for the clinical treatment of bronchial asthma.

## Methods

This study was carried out according to the report items in the PRISMA extended statement of systematic review and network Meta-analysis [10].

This study has not been pre-registered, but was carried out by strictly following the pre-prepared search strategy and study plan.

Software used in this study included Endnote X9, Micro Excel 2019, and Stata 15.1.

### Inclusion and exclusion criteria

#### Type of study

Randomized controlled trials.

#### Subjects

Patients who met the diagnostic criteria for bronchial asthma [1, 2]. No restrictions were imposed on age, gender, or race. The specific diagnostic criteria are as follows:

• The patients have typical asthma symptoms, such as recurrent wheezing, shortness of breath, chest tightness, or coughing, which are not caused by other diseases.
• The patients have a detailed record of asthma or examination of asthma, or the results of pulmonary function/reversibility tests performed during the initial visit to a doctor can support a diagnosis of asthma.

#### Interventions

The intervention in the treatment group was acupuncture, moxibustion, electroacupuncture, ignipuncture, flying needle acupuncture, acupoint catgut embedding, acupoint application, or warm-needle moxibustion. The control group used an acupuncture modality different from the treatment group, or the medications recommended by the guidelines such as inhaled corticosteroids, antihistamines, drugs containing theophylline, and β2 receptor agonists.

The definitions of different interventions are as follows:

Ignipuncture refers to rapidly inserting a warmed-up acupuncture needle into an acupoint or a specific part of the human body to treat diseases.

Electroacupuncture refers to applying microcurrents of different waveforms to stimulate the acupoints after inserting the needles.

Warm-needle moxibustion refers to placing a burning moxa cone on the end of the needle to exert warm stimulation on the body.

Acupoint catgut embedding is to exert therapeutic effects by embedding absorbable sutures materials into the acupoints to perform continuous stimulation.

Acupoint application is a painless acupoint therapy that pastes pharmaceutical powders, which are made by TCM herbs and modulated into pastes with water and oil, on the acupoints to achieve therapeutic effects.

#### Outcome indicators

Primary outcome: total effective rate. Secondary outcomes: FEV1 (Forced expiratory volume in the first second), FVC (Forced vital capacity), ACT score (Asthma control test score), PEF (Peak expiratory flow), IgE (Immunoglobulin E), and incidence of adverse events.

#### Exclusion criteria

①repeated publication; ②incorrect or incomplete data; ③The full-texts unavailable; ④ Non-RCT design, including clinical trials of which the randomization methods were obviously incorrect such as following the order of visits or medical record numbers.

### Literature search strategy

The following databases were searched from the inception of the databases to 26^th^, August 2022: CNKI, Wanfang, Vip (VIP), China Biomedical Database (CBM), PubMed, Embase, Cochrane Library, and Web of Science. The combination of subject headings and free words were applied in literature search, and only Chinese and English studies were searched. The study selection process of PubMed is taken as an example, which is shown in Table 1.

**Table 1:** Pubmed search formula

SEARCH STRATEGY OF PUBMED	
#1	(Asthma[MeSH Terms])
#2	((((Asthma[Title/Abstract]) OR (Asthmas[Title/Abstract])) OR (Bronchial Asthma[Title/Abstract])) OR (Asthma, Bronchial[Title/Abstract]))
#3	#1 OR #2
#4	(((((Acupuncture[MeSH Terms]) OR (Acupuncture Therapy[MeSH Terms])) OR (Acupuncture, Ear[MeSH Terms])) OR (Electroacupuncture[MeSH Terms])) OR (Moxibustion[MeSH Terms]))
#5	((((((((((((((((Acupuncture[Title/Abstract]) OR (Pharmacopuncture[Title/Abstract])) OR (Acupuncture Therapy[Title/Abstract])) OR (Acupuncture Treatment[Title/Abstract])) OR (Acupuncture Treatments[Title/Abstract])) OR (Pharmacoacupuncture Treatment[Title/Abstract])) OR (Pharmacoacupuncture Therapy[Title/Abstract])) OR (Acupotomy[Title/Abstract])) OR (Acupotomies[Title/Abstract])) OR (Acupuncture, Ear[Title/Abstract])) OR (Ear Acupunctures[Title/Abstract])) OR (Auricular Acupuncture[Title/Abstract])) OR (Auricular Acupunctures[Title/Abstract])) OR (Electroacupuncture[Title/Abstract])) OR (Moxibustion[Title/Abstract])) OR (Moxabustion[Title/Abstract]))
#6	#4 OR #5
#7	#3AND #6

### Literature screening and data extraction

Two investigators (Xingyi Wang, Shuyun Zeng) screened the literature using Endnote X9 and extracted data in Excel according to the inclusion and exclusion criteria. If there was any disagreement, a third researcher would be asked to assist in the determination. First, Endnote X9 was used for the initial screening of imported literature; then, the title and abstract were read and checked to exclude literature that did not meet the inclusion criteria. Finally, the full texts of the potentially eligible studies were read to identify studies that could be included in this quantitative analysis. The extracted data included (1) basic information on the included literature: title, author, date of publication; (2) basic characteristics of the included studies: sample size, gender, age, course of the disease, drug dose, course of treatment; (3) outcome indicators; (4) factors of risk of bias: random methods, allocation concealment, and blinding methods.

### Risk of bias assessment

Two reviewers independently completed the risk of bias assessment by using the Cochrane Risk of Bias Assessment Tool 2 (RoB2) [11]. RoB2 contains the following 5 domains: randomization process, deviations from intended interventions, missing outcome data, measurement of the outcome, and selective reporting. Each domain could be graded as “low risk”, “high risk”, or “some concerns”. Quality assessment criteria were as follows: a study with all the domains graded as “low risk” would be considered of high quality; a study with several domains graded as “some concerns” but with no high-risk domain would be considered of moderate quality; and a study with more than 1 domain graded as “high risk” would be considered of low quality. A workable macros EXCEL file published by RoB2 (Revision 2019) [12] was used for the risk of bias assessment.

### Statistical analysis

The network meta-analysis was performed using the mvmeta and network [13, 14] package of Stata 15.1. Dichotomous variables were expressed as relative risk (RR), and continuous variables were reported as weighted mean difference (MD). The 95% confidence interval (CI) was also provided. The fitting inconsistencies test was performed on the closed loops formed in the network meta-analysis. If *P* > 0.05, the inconsistencies in direct and indirect comparisons were not statistically significant, and a consistency model could be used for meta-analysis. If there was no closed loop, the consistency model could be used directly. The interventions of each outcome indicator were ranked by the area under the curve (surface under the cumulative ranking curves, SUCRA), and the SUCRA value was an indicator reflecting the best efficacy possibility of each intervention. The closer the value was to 100%, the better the efficacy of the intervention [15]. A *P* < 0.05 indicated a statistically significant difference. Stata15.1 was used to draw a "comparison-correction" funnel plot, and Egger's test was used to test whether there was a small sample effect in the intervention network and whether there was a publication bias in the included studies.

## Result

### Literature search results

**Fig. 1:**
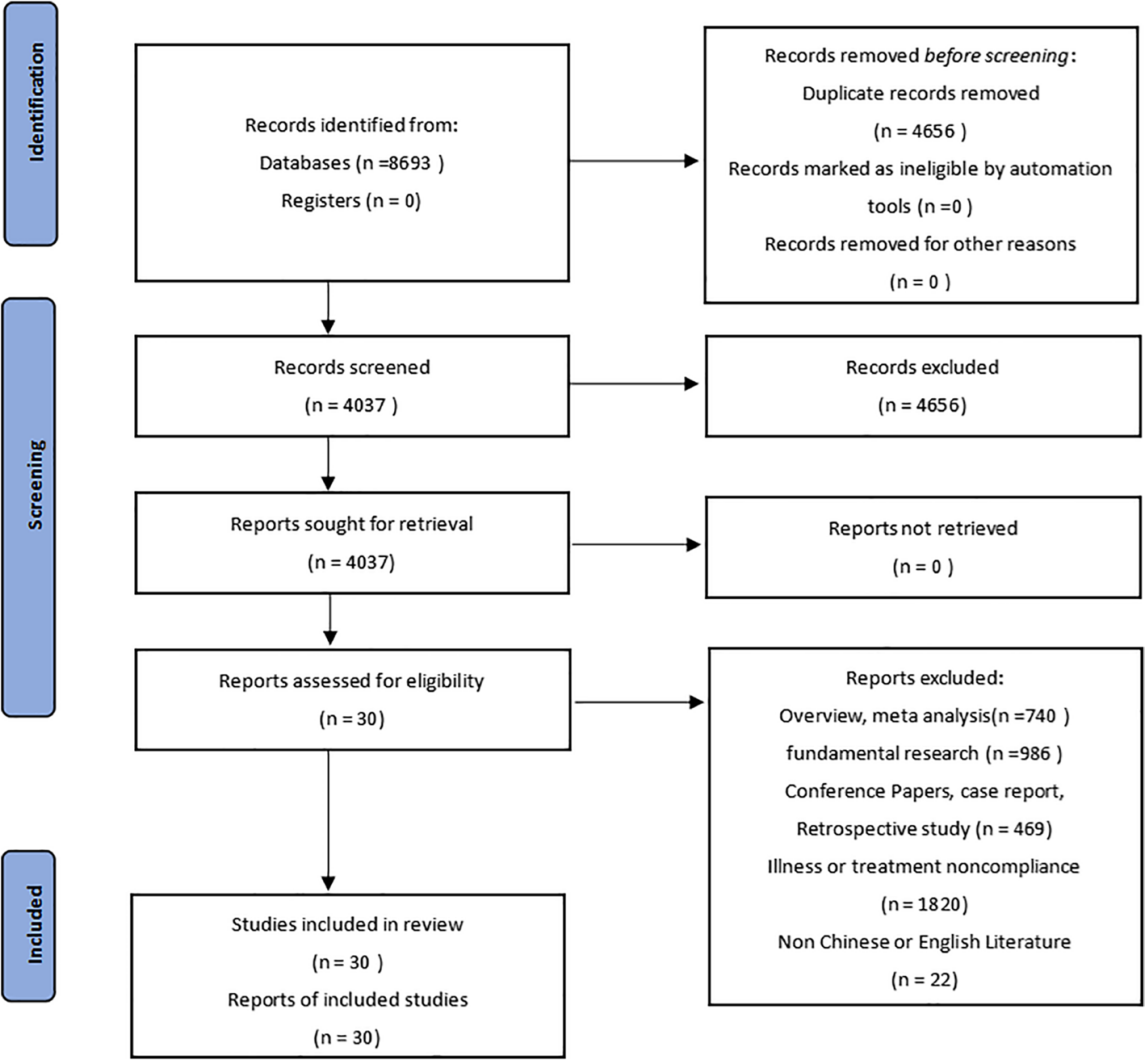
Literature screening process

The preliminary search obtained 8,693 relevant literature, and 3,032 duplicate studies were removed after importing to EndnoteX9. Then, 2,978 articles were removed after reading the titles and abstracts. After a full-text review, 30 articles were finally included [16–45]. Quantitative analysis was performed, and the screening process is shown in Fig. 1. The complete search formulas were provided as a Supplemental file.

### Literature quality evaluation was included

**Fig. 2:**
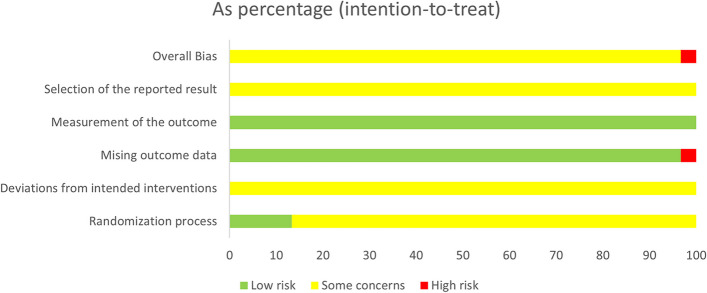
Percentage of projects at risk of bias included in the study

A total of 30 RCTs were finally included, involving 2,722 patients. For the domain of randomization process, only 4 studies (13.3%) were graded as “low risk”, and the others were all of “some concerns”, which might be attributed to the fact that most of the studies failed to provide detailed descriptions of the randomization process and allocation concealment. For the domain of deviations from intended interventions, it was difficult to perform blinding due to the particularity of acupuncture, and all the studies failed to describe the use of proper analysis for estimating the deviations. Therefore, certain risks existed. For the domain of missing outcome data, only 1 study reported dropped-out cases without describing the reasons for dropout, which was graded as “high risk”. The rest of the studies provided almost all data on the participants. For the domain of outcome measurement, all the studies described the outcome-measuring methods and statistical analysis methods, and were all graded as “low risk”. As for the selection of the reported result, all the studies were of “some concerns” due to no specific descriptions of the in-advance registration or publication of the study protocol. Among the 30 studies, 1 study had a high risk of bias and was considered of low quality, and the other 29 studies had certain risks and were of moderate quality. Characteristics of the included studies are shown in Table 2, and detailed risk of bias assessment results are illustrated in Fig. 2.

**Table 2:** Basic characteristics of included study

researcher	Sample size (T/C)	Age/years		Interventions		Treatment course/week	Adverse reactions	Outcome indicators
		T	C	T	C			
Li. Etal 2012 [9]	32/32	41.75 ± 14.71	42. 95 ± 13.40	Moxibustion	Traditional medicine therapy	12		①
Xie. Etal 2015 [17]	90/90	15 ~ 60	15 ~ 60	Acupuncture	Traditional medicine therapy	12.5		①②③
Zhao. Etal 2019 [8]	15/15	40.1 ± 11.7	41.7 ± 13.6	Moxibustion	Traditional medicine therapy	12		①②④
Liang. Etal 2010 [11]	17/19	43.3 ± 12.2	44.4 ± 13.0	Moxibustion	Traditional medicine therapy	12		①②
Ji. Etal 2015 [13]	40/40	42.7 ± 12.1	45.1 + 11.7	Moxibustion	Traditional medicine therapy	12		① ④
Li. Etal 2016 [14]	30/30	55.57 ± 13.67	57.30 ± 13.35	Acupuncture	Traditional medicine therapy	20		①②③④
Xiong. Etal 2014 [16]	55/55	40 ± 20	40 ± 19	Moxibustion	Acupuncture	4		①
Wang. Etal 2012 [10]	30/25	12 ~ 76	14 ~ 75	Moxibustion	Warm needle acupuncture	12.8		①
Wed. etal 2015 [15]	40/40	7.24 ± 4.91	7.93 soil 5.15	Flying needle acupuncture	Acupuncture	0.5		①
Li. Etal 2010 [12]	46/46	49. 68 ± 10. 71	51. 43 ± 10. 56	Electroacupuncture	Traditional medicine therapy	2		①②③
The. Etal 2013 [23]	136/136	15 ~ 84	18 ~ 78	Acupoint application	Acupuncture	24		①
Zhou. Etal 2015 [25]	40/40	61 ± 1.08	59 ± 2.17	Acupoint catgut embedding	Acupuncture	12		①
Zhang. Etal 2017 [24]	40/40	10.64 ± 5.3	11.4 ± 6.07	Acupoint catgut embedding	Traditional medicine therapy	12	There were 1 case of acupuncture fainting and 2 cases of local redness and swelling in the acupoint catgut embedding group, 2 cases of gastrointestinal discomfort, 1 case of insomnia, and 1 case of arrhythmia	① ④⑤
The. Etal 2016 [22]	60/30	4.53 ± 1.52	4.62 ± 1.43	Acupoint application	Traditional medicine therapy	8		①
Hu. Etal 2017 [19]	38/38	39.76 ± 12.15	39.55 ± 12.22	Acupoint catgut embedding	Traditional medicine therapy	12		①
Wang. Etal 2019 [21]	31/31	5.58 ± 2.11	5.58 ± 2.11	Acupoint application	Moxibustion	7		①
Tian. Etal 2020 [20]	30/30	44.5 ± 11.5	44.0 ± 12.5	Acupoint catgut embedding	Traditional medicine therapy	12		①②
Chen. Etal 2020 [18]	80/80	6.02 ± 2.74	5.97 ± 2.68	Acupoint application	Moxibustion	7		①
Yu. Etal 2004 [26]	45/45	18 ~ 63	19 ~ 61	Ignipuncture	Traditional medicine therapy	4	Five patients in the fire needle group had itching at the acupoint, and 7 patients in the control group had Candida albicans stomatitis and hoarseness	① ⑤
Sun. etal 2011 [27]	30/30	19 ~ 65	19 ~ 65	Acupoint application	Traditional medicine therapy	12		①②
Yao. Etal 2010 [28]	30/30	48.10 ± 15.19	47.30 ± 12.30	Acupoint catgut embedding	Traditional medicine therapy	12		①
Tian.etal 2013 [29]	50/50	49 ± 15	51 ± 13	Acupoint application	Electroacupuncture	144		①
Li. Etal 2005. [30]	30/28	35 ~ 74	24 ~ 65	Electroacupuncture	Traditional medicine therapy	2		①
Ou. Etal. 2011 [33]	28/29	45.13 ± 7.8	15.52 ± 9.6	Moxibustion	Traditional medicine therapy	12		①②
Yi. Etal. 2020 [31]	99/99	9.34 ± 2.34	9.57 ± 2.42	Moxibustion	Traditional medicine therapy	1		①
Huo. etal. 2016 [32]	45/45	39.67 ± 12.34	38.92 ± 11.79	Acupuncture	Traditional medicine therapy	8		②③
Shu. etal. 2017 [34]	60/60	44.23 ± 11.12	45.11 ± 10.22	Acupoint application	Traditional medicine therapy	4	Disinfection treatment for larger blisters	①②③⑤
Song. etal. 2012 [35]	32/32	46.0 ± 15.3	46.0 ± 12.1	Moxibustion	Traditional medicine therapy	12		①
Zhao.etal. 2010 [36]	37/31	40.12 ± 9.95	40.12 ± 9.95	Acupoint catgut embedding	Acupuncture	24		①
Zhang. Etal. 2005 [37]	60/30	27.4 ± 9.2	26.8 ± 8.7	Acupuncture	Traditional medicine therapy	1		①

(1): Clinical effective rate (2): FEV1 (3): FVC (4): ACT scores (5): Adverse reactions

### Quality assessment of the evidence of the included studies

The quality of evidence of the included studies was evaluated according to the GRADE assessment method, and seven outcome indicators were analyzed. Evidence for the total effective rate, FEV1, FVC and ACT scores was of medium quality. The quality of evidence for PEF, IgE, and adverse reactions was low. The decline in quality was mainly caused by the limitations of randomization method and the blinding method as well as the imprecision due to the small sample size. The detailed quality assessment is shown in Table 3.

**Table 3:** The GRADE assessment of outcome indicators

outcome indicator	Downgrading factors					Quality Grade
	boundedness	Inconsistency	Not directly	Inaccuracy	Publication bias	
Total effective rate	-1	0	0	0	0	middle rank
*FEV*_*1*_	-1	0	0	0	0	middle rank
FVC	-1	0	0	0	0	middle rank
ACT scores	-1	0	0	0	0	middle rank
PEF	-1	0	0	0	-1	low rank
IgE	-1	0	0	-1	0	low rank
Adverse reactions	-1	0	0	-1	0	low rank

### Network meta-analysis

#### Total effective rate

**Fig. 3:**
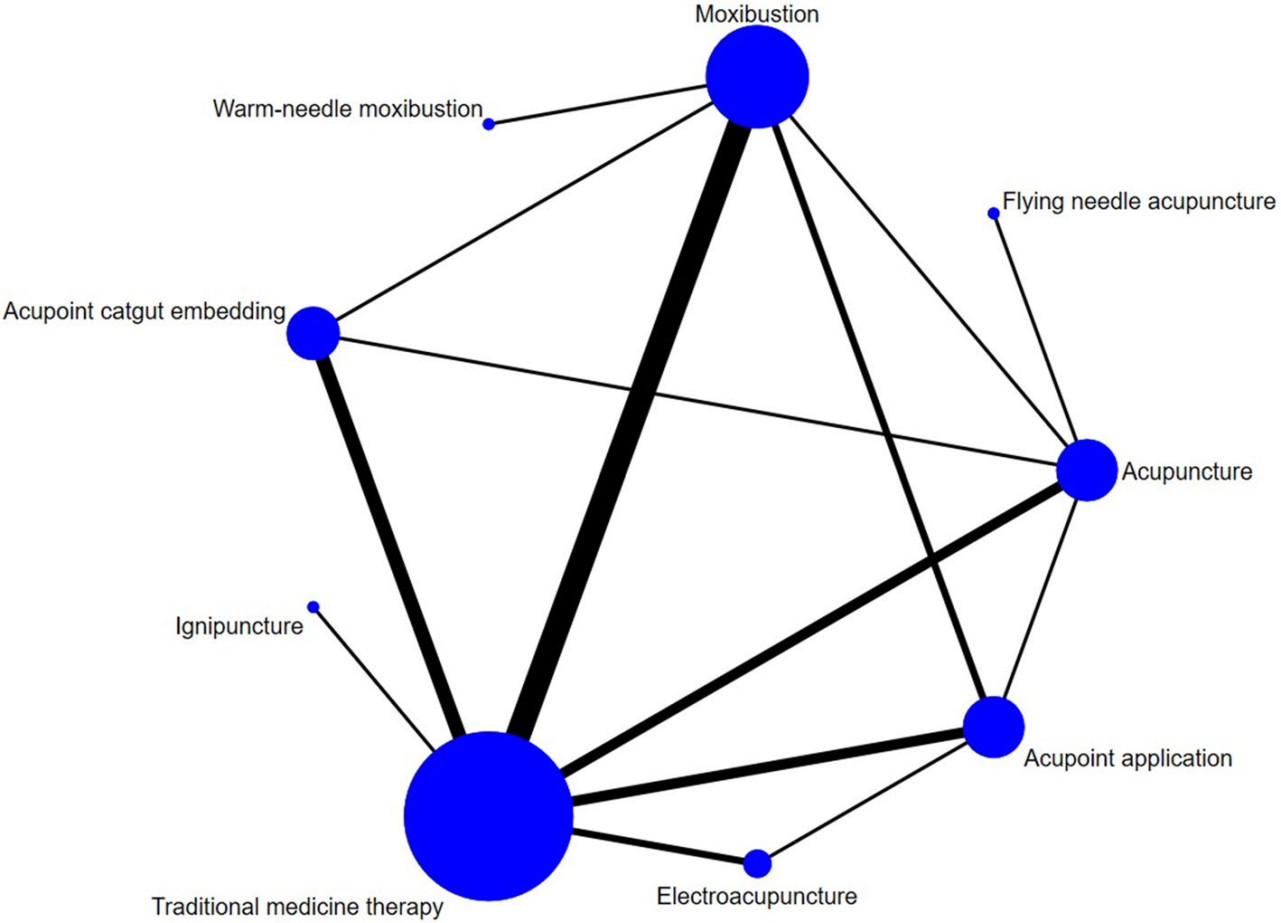
Total effective rate network diagram

There were 29 studies reporting the total effective rate, involving 9 interventions. Most of the studies compared moxibustion with Traditional medicine therapy. The network evidence diagram is presented in Fig. 3. A closed loop was observed so that inconsistency test was performed, and the results showed that the consistency was good between the direct and indirect comparisons (*p* > 0.05). Network meta-analysis should be performed based on the consistency model. The SUCRA probability ranking is shown in Table 4. Acupoint application was the most likely to become the most effective intervention. The probability ranking was: Acupoint application > acupoint catgut embedding > ignipuncture > flying needle acupuncture > electroacupuncture > moxibustion > warm-needle moxibustion > manual acupuncture = Traditional medicine therapy. The league table is shown in Table 5.

**Table 4:** SUCRA probability ranking

Interventions	Total effective rate		FEV_1_		FVC		ACT scores		PEF		IgE	
	SUCRA%	ranking	SUCRA%	ranking	SUCRA%	ranking	SUCRA%	ranking	SUCRA%	ranking	SUCRA%	ranking
A	35.7	8	77.1	2	59.5	3	45.1	2	45.1	2	54.2	2
B	57.9	6	18.8	5	-	-	16.4	4	16.4	4	19.5	4
C	32.6	5	49.3	3	38.7	2	-	-	-	-	-	-
D	73.1	3	-	-	-	-	-	-	-	-	**93.1**	**1**^**a**^
E	51.3	4	-	-	-	-	-	-	-	-	-	-
F	76	2	56.4	4	-	-	**95.8**	**1**^**a**^	**95.8**	**1**^**a**^	32.4	3
G	**81.7**	**1**^**a**^	**82.9**	**1**^**a**^	**91.7**	**1**^**a**^	-	-	-	-	-	-
H	17.9	7	-	-	-	-	-	-	-	-	-	-
I	23.8	8	15.6	6	10.1	4	42.8	3	42.8	3	72.7	5

A: Acupuncture B: Moxibustion C: Electroacupuncture D: Ignipuncture E: Flying needle acupuncture F: Acupoint catgut embedding G: Acupoint application H: Warm needle acupuncture I: Traditional medicine therapy

^1^^a^The most likely best intervention measure

**Table 5:** Network meta-analysis effective rate

Traditional medicine therapy								
1.12 (0.65,1.94)	Warm needles							
0.87 (0.63,1.21)	0.77 (0.43,1.39)	Acupoint application						
0.73 (0.48,1.09)	0.65 (0.34,1.22)	0.83 (0.53,1.32)	Acupoint catgut embedding					
0.86 (0.59,1.25)	0.77 (0.42,1.42)	0.99 (0.65,1.52)	1.19 (0.73,1.94)	Flying needles				
0.73 (0.51,1.05)	0.65 (0.34,1.26)	0.84 (0.52,1.38)	1.01 (0.59,1.74)	0.85 (0.51,1.43)	Fire needles			
0.97 (0.62,1.52)	0.87 (0.45,1.68)	1.12 (0.83,1.52)	1.34 (0.78,2.32)	1.13 (0.67,1.91)	1.33 (0.75,2.36)	Electroacupuncture		
0.82 (0.57,1.19)	0.73 (0.49,1.11)	0.95 (0.62,1.44)	1.14 (0.70,1.84)	0.96 (0.61,1.51)	1.12 (0.67,1.88)	0.85 (0.50,1.42)	Moxibustion	
1.24 (0.77,2.00)	1.30 (0.90,1.87)	1.04 (0.58,1.89)	1.09 (0.79,1.51)	1.28 (0.85,1.92)	0.97 (0.64,1.45)	1.10 (0.67,1.83)	1.14 (0.83,1.56)	Acupuncture

#### *FEV*_*1*_

**Fig. 4:**
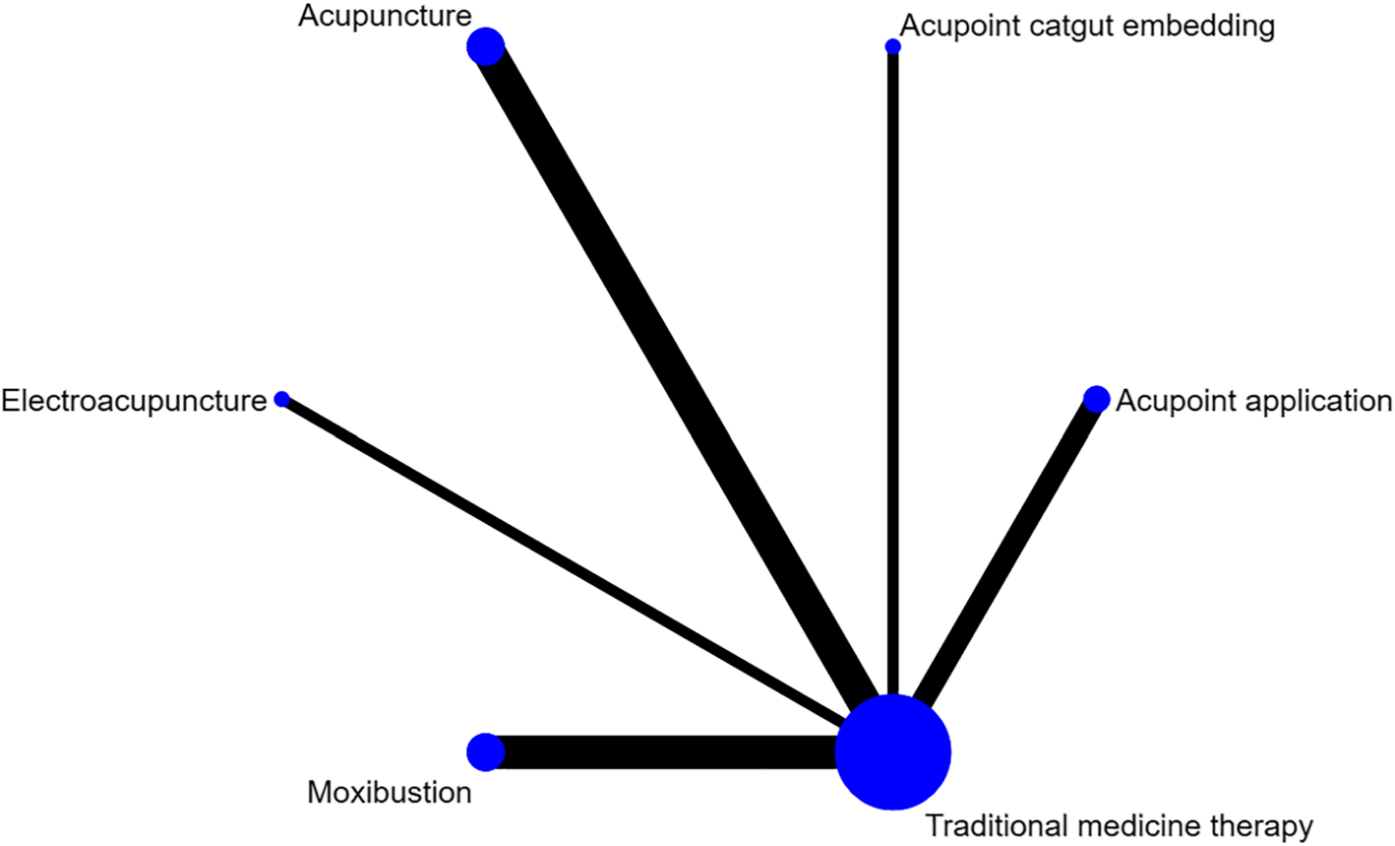
FEV1 network diagram

Ten studies reported FEV_1_, involving acupuncture, moxibustion, electroacupuncture, acupoint catgut embedding, acupoint application and traditional medicine therapy, and no closed loop was formed. Figure 4 shows the network evidence map. Since the pairwise comparisons of all interventions were indirect, there was no need for inconsistency testing, and a consistency model was directly used for analysis. The SUCRA probability ranking results (Table 4) showed that acupoint application had the greatest possibility of becoming the best intervention, and the probability ranking was: acupoint application > acupuncture > electroacupuncture > acupoint catgut embedding > moxibustion > traditional medicine therapy. Of the 15 pairwise comparisons in the league table generated by the network meta-analysis, 4 were statistically significant (*P* < 0.05), including Traditional medicine therapy-acupoint application [-7.29 (-12.11, -2.47)]; Traditional medicine therapy-manual acupuncture [-6.41 (-9.80, -3.01)]; acupoint application-moxibustion [7.20 (0.28,14.11)], and moxibustion-manual acupuncture [-6.32 (-11.89, -0.75)]. Acupoint application would be the optimal intervention in improving the FEV_1_.The league table is presented in Table 6.

**Table 6:** Network meta-analysis of FEV1

Traditional medicine therapy					
**-7.29 (-12. 11,-2.47)**^**a**^	Acupoint application				
-4.26 (-9.55,1.03)	3.03 (-4.13,10.18)	Acupoint catgut embedding			
-3.39 (-13.12,6.34)	3.90 (-6.96,14.75)	0.87 (-10.21,11.95)	Electroacupuncture		
-0.09 (-4.68,4.50)	**7.20 (0.28,14. 11) **^**a**^	4.17 (-2.83,11.17)	3.30 (-7.46,14.06)	Moxibustion	
**-6.41 (-9. 80,-3.01)**^**a**^	0.88 (-5.08,6.84)	-2.15 (-8.43,4.14)	-3.02 (-13.32,7.29)	**-6.32 (-11. 89,-0.75)**^**a**^	Acupuncture

^a^represent statistically significant

#### FVC

**Fig. 5:**
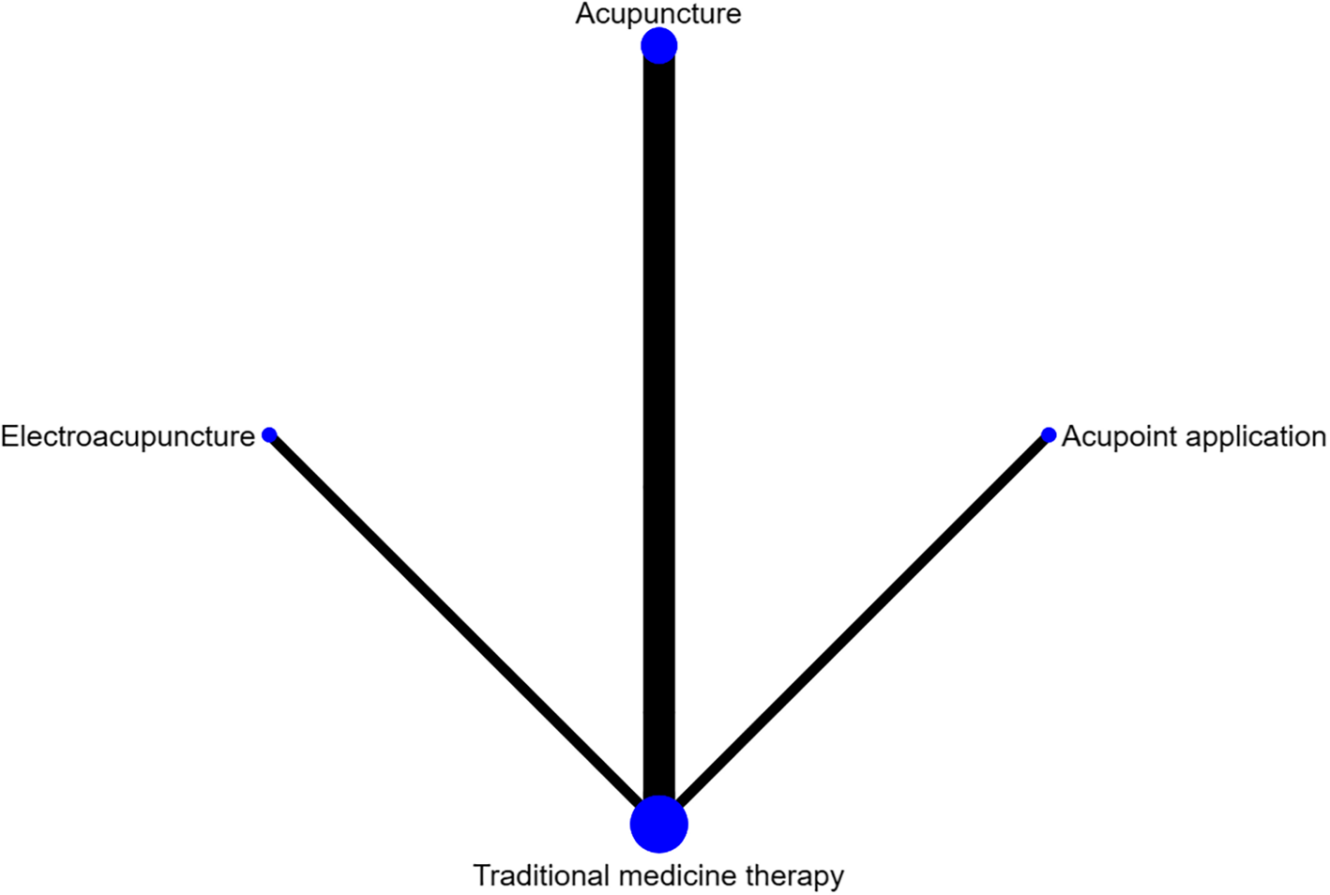
FVC network diagram

Five studies reported FVC, involving acupuncture, electroacupuncture, acupoint application, and traditional medicine therapy, and no closed loop was formed. Figure 5 shows the network evidence map. Since the pairwise comparisons of all interventions were indirect, there was no need for inconsistency testing, and the consistency model was directly used for analysis. The SUCRA probability ranking results (Table 4) showed that acupoint application has the greatest possibility of becoming the best intervention, and the probability ranking was: acupoint application > electroacupuncture > acupuncture > traditional medicine therapy. Of the 3 pairwise comparisons in the league table generated by the network meta-analysis, 2 comparisons were statistically significant (*P* < 0.05), including acupoint application-Traditional medicine therapy [8.02 (2.54,13.50)] and manual acupuncture-Traditional medicine therapy [4.18 (1.10,7.26)]. Acupoint application would be the optimal intervention in improving the FVC. The league table is presented in Table 7.

**Table 7:** Network meta-analysis of FVC

Traditional medicine therapy	8.02 (2.54,13. 50)^a^	2.25 (-6.10,10.60)	4.18 (1.10,7. 26)^a^
	Acupressure point application	-5.77 (-15.76,4.22)	-3.84 (-10.12,2.44)
		Electroacupuncture	1.93 (-6.97,10.83)
			Acupuncture

^a^represent statistically significant

#### ACT scores

**Fig. 6:**
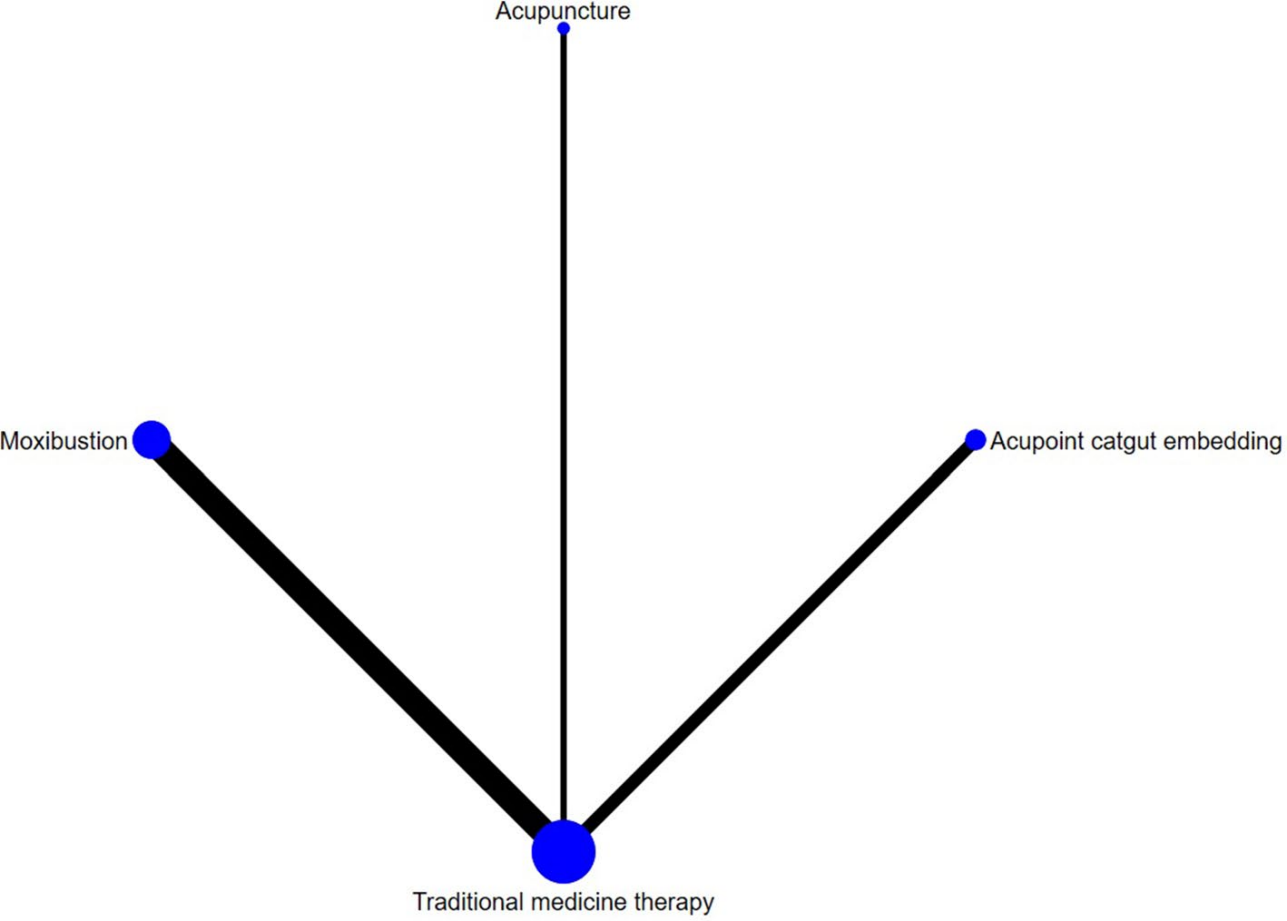
ACT scores network diagram

Seven studies reported ACT scores, involving acupuncture, moxibustion, acupoint catgut embedding, and traditional medicine therapy, and no closed loop was formed. Figure 6 is a network evidence map. Since the pairwise comparisons of all interventions were indirect, there was no need for inconsistency testing, and the consistency model was directly used for analysis. The SUCRA probability ranking results (Table 4) showed that acupoint catgut embedding had the greatest possibility of becoming the best intervention, and the probability ranking was: acupoint catgut embedding > acupuncture > traditional medicine therapy > moxibustion. Among the 6 pairwise comparisons in the league table generated by the network meta-analysis, 2 were statistically significant (*P* < 0.05), including Traditional medicine therapy-acupoint catgut embedding [-4.29 (-7.94, -0.65)] and acupoint catgut embedding-moxibustion [5.52 (1.05,9.99)]. Acupoint catgut embedding would be the most effective intervention in improving the ACT score of asthma patients. The league table is presented in Table 8.

**Table 8:** Network meta-analysis of ACT scores

Traditional medicine therapy			
**-4.29 (-7. 94,-0.65)**^**a**^	Acupoint catgut embedding		
1.23 (-1.37,3.82)	**5.52 (1.05,9. 99)**^**a**^	Moxibustion	
-0.29 (-5.41,4.83)	4.00 (-2.28,10.29)	-1.52 (-7.26,4.22)	Acupuncture

^a^represent statistically significant

#### PEF

**Fig. 7:**
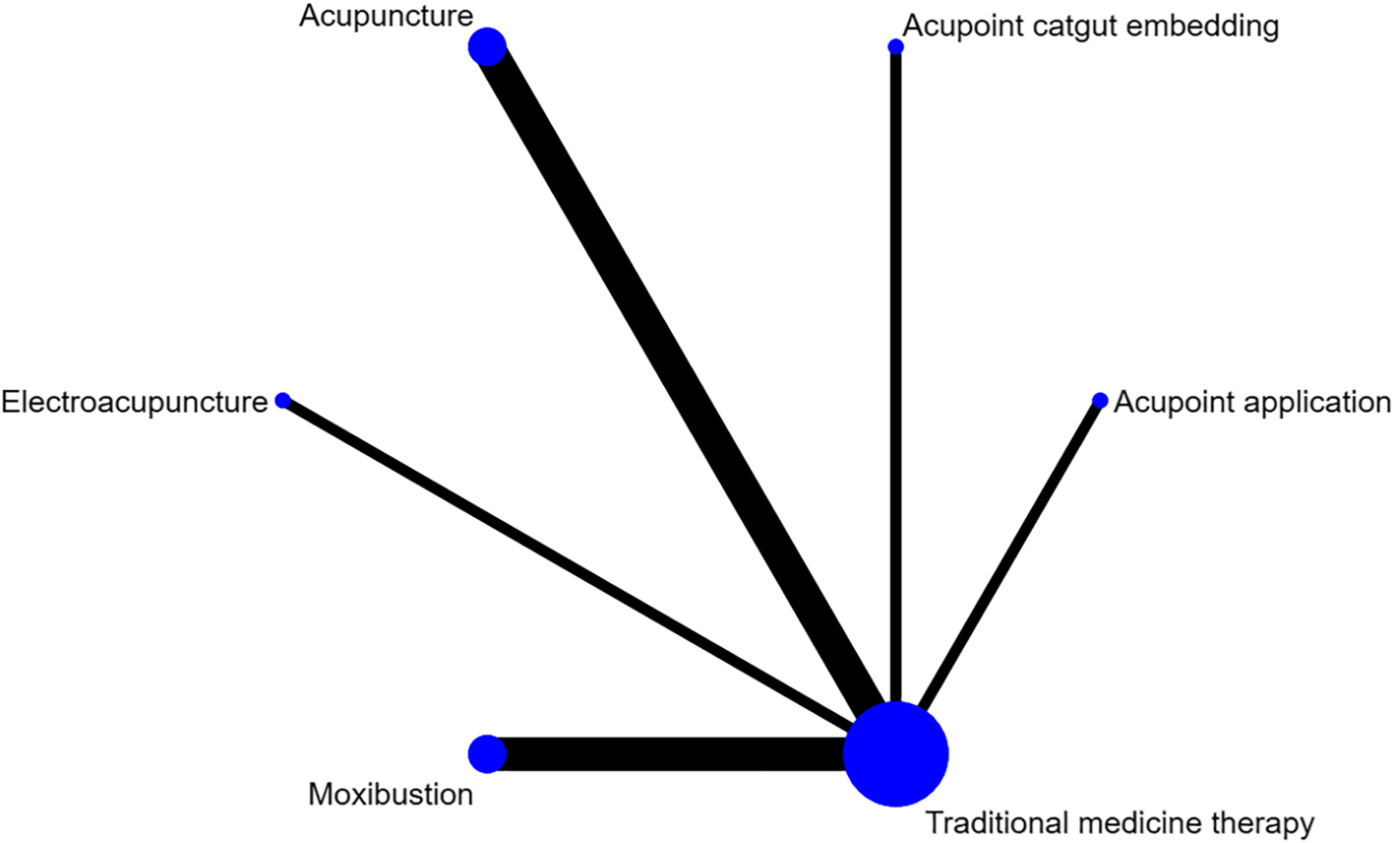
PEF network diagram

Nine studies reported PEF, involving acupuncture, moxibustion, electroacupuncture, acupoint catgut embedding, acupoint application and traditional medicine therapy, and no closed loop was formed. Figure 7 shows the network evidence map. Since the pairwise comparisons of all interventions were indirect, there was no need for inconsistency test, and a consistency model was directly used for analysis. The SUCRA probability ranking results (Table 4) showed that acupoint application had the greatest possibility of becoming the best intervention, and the probability ranking was: acupoint application > electroacupuncture > acupoint catgut embedding > moxibustion > acupuncture > traditional medicine therapy. All the 15 pairwise comparisons in the league table generated by the network meta-analysis were not statistically significant. The results showed that there was no statistical difference between acupuncture and traditional medicine therapy in improving PEF in asthma patients. The league table is presented in Table 9.

**Table 9:** Network meta-analysis of PEF

Traditional medicine therapy					
-8.99 (-23.49,5.51)	Acupoint application				
-3.77 (-16.63,9.09)	5.22 (-14.16,24.60)	Acupoint catgut embedding			
-5.64 (-21.67,10.39)	3.35 (-18.26,24.96)	-1.87 (-22.42,18.68)	Electroacupuncture		
-3.47 (-12.44,5.51)	5.52 (-11.53,22.57)	0.30 (-15.38,15.98)	2.17 (-16.20,20.54)	Moxibustion	
-3.15 (-10.68,4.39)	5.84 (-10.50,22.18)	0.62 (-14.28,15.53)	2.49 (-15.22,20.20)	0.32 (-11.38,12.02)	Acupuncture

#### IgE

**Fig. 8:**
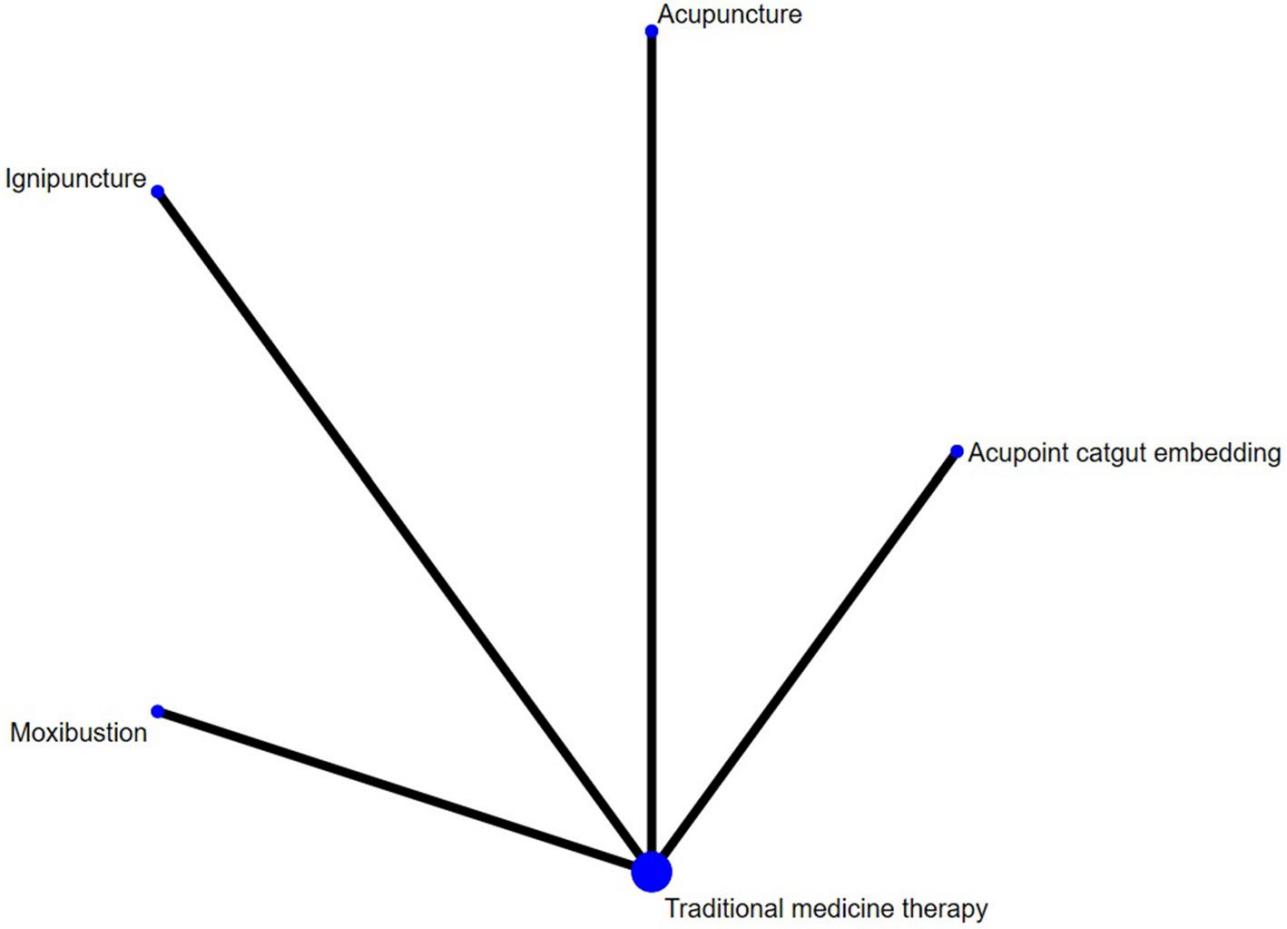
IgE network diagram

Four studies reported IgE, involving acupuncture, moxibustion, ignipuncture, acupoint catgut embedding and traditional medicine therapy, and no closed loop was formed. Figure 8 shows the network evidence map. Since the pairwise comparisons of all interventions were indirect, there was no need to for inconsistency test, and a consistency model is directly used for analysis. The SUCRA probability ranking results (Table 4) showed that ignipuncture had the greatest possibility of becoming the best intervention, and the probability ranking was: ignipuncture > acupuncture > acupoint catgut embedding > moxibustion > traditional medicine therapy. Three of the ten pairwise comparisons in the league table generated by the network meta-analysis were statistically significant (*P* < 0.05), and the results showed that in terms of improving IgE in patients with bronchial asthma, ignipuncture is superior to traditional medicine therapy [95.79 (60.59,130.99)], moxibustion is superior to traditional medicine therapy [49.16 (21.68,76.64)], moxibustion is superior to ignipuncture [-46.63 (-89.64,-3.62)]. The league table is presented in Table 10.

**Table 10:** Network meta-analysis of IgE

Traditional medicine therapy				
40.00 (-6.88,86.88)	Acupoint application			
95.79 (60.59,130.99)^*^	55.79 (-1.82,113.40)	Warm needles		
49.16 (21.68,76.64)^*^	9.16 (-43.91,62.23)	-46.63 (-89.64,-3.62)^*^	Moxibustion	
39.89 (-5.94,85.72)	-0.11 (-69.51,69.29)	-55.90 (-118.26,6.46)	-9.27 (-68.31,49.77)	Acupuncture

#### Adverse reactions

There were 2 studies [26, 34] that reported the occurrence of adverse events, involving ignipuncture, acupoint catgut embedding, and Traditional medicine therapy. Acupuncture-induced adverse events included acupuncture syncope, itching around the inserting points, and swelling of the inserting points. These adverse events disappeared after discontinuation of the treatment. The adverse events of the Traditional medicine therapy group were gastrointestinal discomforts, insomnia, arrhythmia, hoarseness and candida albicans infection. Therefore, acupuncture-induced adverse events seemed to be less severe than those induced by Traditional medicine therapy.

### Publication bias and small sample effect estimation

**Fig. 9:**
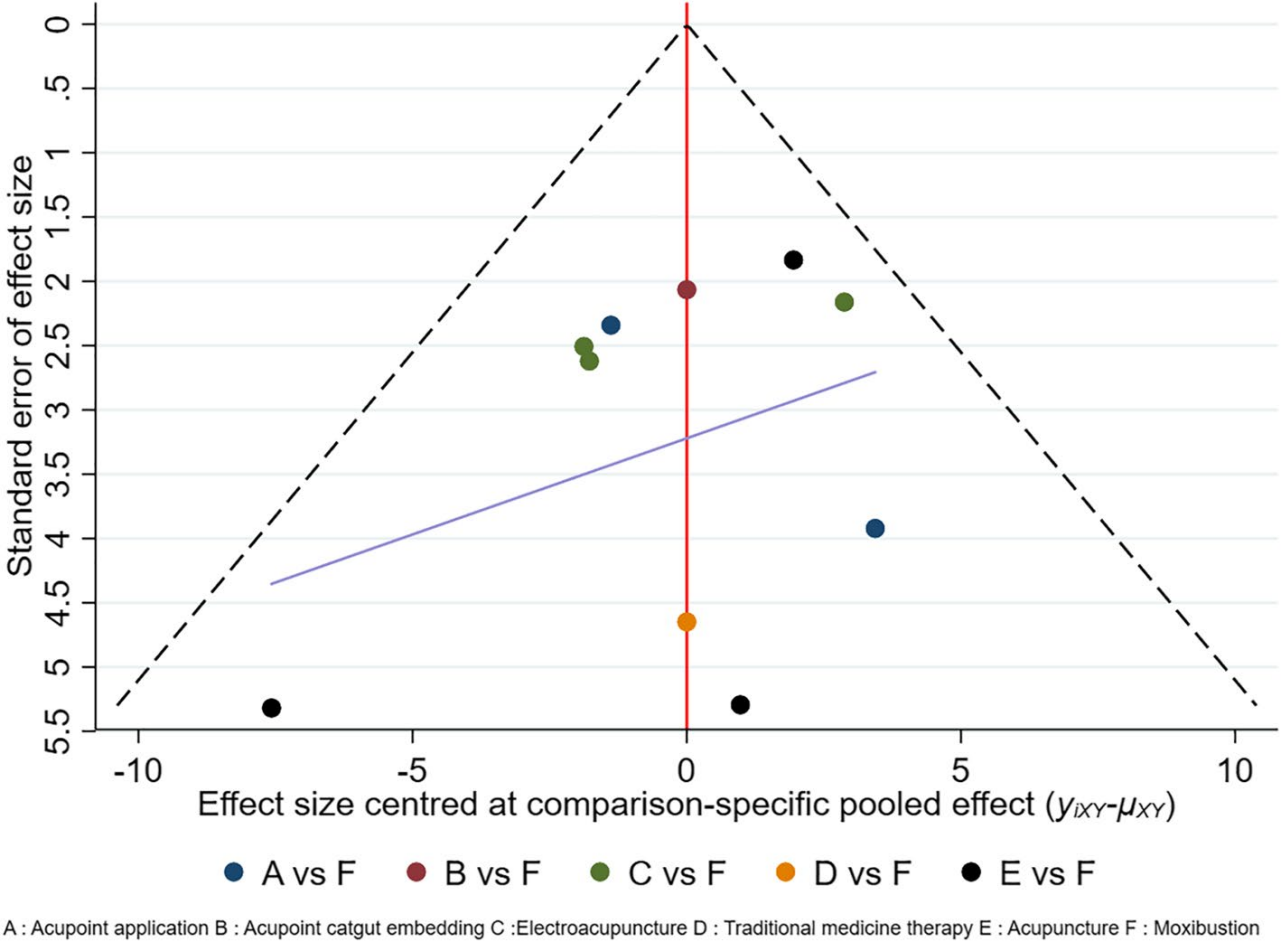
"Compare-correct" funnel of total effective rate

**Fig. 10:**
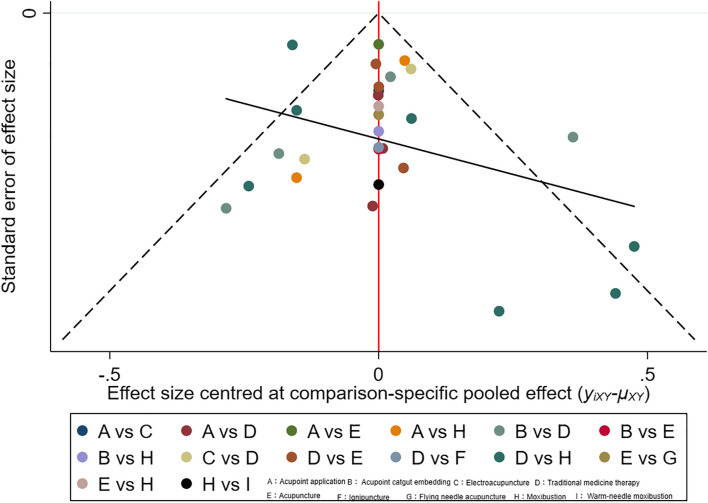
“Compare-correct” funnel plot of FEV1

The 'bias-correction' funnel plot for this network meta-analysis was generated using Stata 15.1 software. Due to the small number of studies for other outcome indicators, only the publication bias and small sample effect of the total effective rate and FEV1 were tested. Figure 9 shows the total effective rate funnel plot. Most of the scattering spots are located in the middle and upper part of the inverted triangle, and their distribution is symmetrical, indicating no publication bias. However, there are still 3 spots dispersing outside of the funnel and one spot scattering at the bottom of the funnel, indicating small sample effects. The funnel plot of the FEV1 is shown in Fig. 10. Most of the scattered points were located in the middle and upper part of the inverted triangle, and the distribution was symmetrical, suggesting no significant publication bias. One scatter point was located at the bottom of the funnel plot, indicating small-sample effects. Egger`s test was performed for the total effective rate and FEV1. The *P* value of the total effective rate was 0.075 > 0.05, and the *P* value of FEV1 was 0.513 > 0.05, indicating small possibility of publication bias in total effective rate and FEV1.

## Discussion

The results of this study indicate that acupoint application is the most effective intervention for improving the total effective rate and pulmonary function. In improving the ACT score of the patients, acupoint catgut embedding presents to be the optimal intervention. Ignipuncture is the optimum intervention to reduce IgE level. We searched eight Chinese and English databases and evaluated 7 aspects of the 9 therapies including acupuncture, moxibustion, electroacupuncture, ignipuncture, flying needle acupuncture, acupoint catgut embedding, acupoint application, warm needle acupuncture, and traditional medicine therapy. The outcome indicators contained the total effective rate, FEV_1_, FVC, ACT scores, and incidence of adverse reactions. The above 9 therapies for the treatment of bronchial asthma were compared and analyzed. traditional medicine therapy can effectively control and relieve the symptoms of bronchial asthma, but it has some adverse reactions and can bring a heavy financial burden to patients [46]. Therefore, it is significant to explore effective alternative treatments for bronchial asthma. Acupuncture features few adverse reactions, low cost, and simple operation, which makes it broadly accepted by patients with bronchial asthma. At present, multiple fundamental studies, clinical studies and meta-analyses have verified the efficacy and safety of acupuncture in the treatment of bronchial asthma. Acupuncture can treat bronchial asthma by controlling the inflammatory response, preventing airway remodeling, improving lung ventilation, improving cellular immune function, and regulating the neuroendocrine network [47]. However, few studies have compared the effects of different acupuncture methods in the treatment of bronchial asthma. Therefore, it is important to explore the most suitable acupuncture method as an alternative therapy for the clinical treatment of bronchial asthma. Network meta-analysis can be used to analyze multiple outcome indicators of multiple acupuncture therapies in the treatment of bronchial asthma and provide evidence-based medical evidence for the clinical treatment of bronchial asthma [10].

There have been multiple studies demonstrating that the therapeutic effect of acupoint application in asthma treatment could be associated with the readjustment of Th1/Th2 and the up-regulation of serum lipoxin A4 (LXA4), peripheral eosinophils (EOS), and serum S1P expression. It could also improve the pulmonary and immune function of the patients [48]. It is conventionally believed that acupoint application would be more effective in winter or summer. It could also be used at any time to treat bronchial asthma on a daily basis [9]. Acupoint application is a painless and heat-free treatment. Compared with other treatments that are invasive or exert thermal stimulation, acupoint application causes less pain, so the patients are easy to adhere and the more compliant. Furthermore, the materials of acupoint application are easy to make, with low treatment costs. It brings a small economic burden on the patients, making them easy to adhere to the treatment. Therefore it can be clinically recommended as a complementary and alternative therapy for bronchial asthma.

Acupoint catgut embedding reduces EOS aggregation and promotes its regulation to alleviate the airway inflammatory response in the episode of asthma, which might be associated with the inhibition of p38MAP, ICAM-1, and IL-4 mRNA expression [49]. Acupoint catgut embedding is a long-acting acupuncture therapy, which avoids distress caused by short-term and high-frequency acupuncture. Moreover, there have been certain research basis in the embedding tools, sutures, and treatment regimens of acupoint catgut embedding. With the development of modern chemical technology, the embedded suture has been able to self-degrade, with no rejection reaction, and it is developing into a characteristic direction, such as expansive materials, self-heating materials, intelligent materials, etc. [50]. The disposable embedding needle is the mainly-used embedding tool, and its development direction is mainly automation and being painless [51].

The GINA guideline recommends drug therapies like ICS-formoterol or SABA, but the patient compliance to ICS is poor, leading to an increased risk of deterioration of disease [1]. In this case, acupuncture-related therapies can be used as alternative treatments to control the disease. In the compilation of the guide, I suggest that acupuncture and moxibustion related therapies should be included in the supplementary treatment methods.Among the existing medical guidelines on bronchial asthma, only the Chinese guidelines include acupuncture and moxibustion therapies.In clinical practice, acupuncture and moxibustion related therapy can be used as an alternative to medication.Especially for patients who experience adverse reactions or poor adherence to traditional drugs.In terms of economy, acupuncture and moxibustion related therapies have the advantage of being cheap compared with traditional medication.Cheap therapies also benefit patients' adherence to treatment.At the same time, acupuncture therapy can also be combined with medication, so as to achieve better outcomes.

However, this study has several limitations: ① No standardized inclusion and exclusion criteria were set for study selection, which might cause bias. ② The included studies failed to provide detailed information on the randomization process and allocation concealment. ③ Only 1 of the included studies performed blinding. It is difficult to implement blinding of participants and personnel due to the particularity of acupuncture therapy, while blinding of outcome assessment should be performed as far as possible to avoid bias. ④The included studies possibly had no pre-registration and pre-published study protocol. This could cause the authors to selectively report the positive results. ⑤ Acupuncture therapy has many differences in the acupoint selection, manipulation, and frequency, which could cause heterogeneity. Well-designed subgroup analysis should have been conducted. ⑥ Despite the large number of RCTs regarding acupuncture therapies for the treatment of asthma, there remains a lack of high-quality studies. Several studies have compared different acupuncture modalities, while these studies usually recruited few participants.⑦ More effective assessment tools such as CINeMA should be used to evaluate the quality of the included studies [52].⑧ Normalized entropy is an alternative tool for measuring the uncertainty of treatment ranking by improving the translation of results from NMAs to clinical practice and avoiding naïve interpretation of treatment ranking [53].

For future studies, more precise investigation into the different acupuncture therapies should be a focus for the researchers. Although there are many studies on the mechanism and clinical observation of acupuncture for the treatment of asthma, the acupuncture therapy involving different selection of acupoints, depth of acupuncture, time of needle retention and frequency of treatment was rarely explored from the perspective of the efficacy of selection of acupoints and different operation methods for bronchial asthma, which should be focused on in future clinical research to find out the regularity of selection of acupoint and acupuncture method in the treatment of bronchial asthma, and to provide theoretical basis of acupuncture for the clinical treatment of bronchial asthma. In addition, there are a variety of studies on the mechanism of acupuncture, including signaling pathways and cytokines, but most of the signaling pathways-based studies were carried out using the nodal signaling pathways as the study subjects, with a lack of continuity and integrity, and the mechanisms of some signaling pathways related to acupuncture for the treatment of asthma have not been clearly described, without sufficient study breadth and depth, or even have not been reported in any previous study. In future studies, researchers should combine proteomics and genomics theories and technologies to further clarify the specific regulatory mechanism and characteristics of acupuncture in the treatment of asthma, and find new clues for the treatment of asthma through animal studies of acupuncture.

## Conclusion

The limitations of this study remind us that the following aspects should be addressed in future clinical practice: a consensus should be reached among clinicians and researchers to unify the inclusion criteria for patient recruitment, such as the unified use of GINA diagnostic criteria. Clinical researchers should design the study protocol and report the results in strict accordance with the CONSORT principle. The study protocol should be registered on the well-accepted registration platforms before conducting the trial. More well-designed, large-sample, and multi-centric clinical trials and more comprehensive meta-analyses are needed to further validate and supplement our findings.

In general, Different acupuncture and moxibustion Therapy has advantages in terms of costs, operation difficulty, and patient compliance. They can be used as a complementary and alternative therapy, together with pharmaceutical gents, for the treatment of bronchial asthma.

### Supplementary Information

**Additional file 1.** 

**Additional file 2.**

## Data Availability

The original contributions presented in the study are included in the article, further inquiries can be directed to the corresponding author.
