# Supplementary material for: A network meta-analysis of different acupuncture modalities in the treatment of bronchial asthma
Source: BMC Pulm Med. 2023 Sep 22;23:357. doi: 10.1186/s12890-023-02645-8 (PMC10517502; doi:10.1186/s12890-023-02645-8)
Supplement: Supplementary file 1 — Additional file 1. [file 12890_2023_2645_MOESM1_ESM.docx]

Supplementary material: search strategy

| **SEARCH STRATEGY OF PUBMED** | |
| --- | --- |
| #1 | (Asthma[MeSH Terms]) |
| #2 | ((((Asthma[Title/Abstract]) OR (Asthmas[Title/Abstract])) OR (Bronchial Asthma[Title/Abstract])) OR (Asthma, Bronchial[Title/Abstract])) |
| #3 | #1 OR #2 |
| #4 | (((((Acupuncture[MeSH Terms]) OR (Acupuncture Therapy[MeSH Terms])) OR (Acupuncture, Ear[MeSH Terms])) OR (Electroacupuncture[MeSH Terms])) OR (Moxibustion[MeSH Terms])) |
| #5 | ((((((((((((((((Acupuncture[Title/Abstract]) OR (Pharmacopuncture[Title/Abstract])) OR (Acupuncture Therapy[Title/Abstract])) OR (Acupuncture Treatment[Title/Abstract])) OR (Acupuncture Treatments[Title/Abstract])) OR (Pharmacoacupuncture Treatment[Title/Abstract])) OR (Pharmacoacupuncture Therapy[Title/Abstract])) OR (Acupotomy[Title/Abstract])) OR (Acupotomies[Title/Abstract])) OR (Acupuncture, Ear[Title/Abstract])) OR (Ear Acupunctures[Title/Abstract])) OR (Auricular Acupuncture[Title/Abstract])) OR (Auricular Acupunctures[Title/Abstract])) OR (Electroacupuncture[Title/Abstract])) OR (Moxibustion[Title/Abstract])) OR (Moxabustion[Title/Abstract])) |
| #6 | #4 OR #5 |
| #7 | #3AND #6 |

| SEARCH STRATEGY OF COCHRANE | |
| --- | --- |
| #1 | (Acupuncture OR Pharmacopuncture):ti, ab, kw |
| #2 | (Acupuncture Therapy OR Acupuncture Treatment OR Acupuncture Treatments OR Pharmacoacupuncture Treatment OR Pharmacoacupuncture Therapy ):ti, ab, kw |
| #3 | (Acupotomy OR Acupotomies):ti, ab, kw |
| #4 | (Acupuncture, Ear OR Ear Acupunctures OR Auricular Acupuncture OR Auricular Acupunctures):ti, ab, kw |
| #5 | (Electroacupuncture):ti, ab, kw |
| #6 | (Moxibustion OR Moxabustion):ti, ab, kw |
| #7 | #1 OR #2 OR #3 OR #4 OR #5 OR #6 |
| #8 | (Asthma OR Asthmas OR Bronchial Asthma OR Asthma, Bronchial):ti, ab, kw |
| #9 | #7 AND #8 |

| SEARCH STRATEGY OF EMBASE | |
| --- | --- |
| #1 | (Acupuncture OR Pharmacopuncture):ab,ti |
| #2 | (Acupuncture Therapy OR Acupuncture Treatment OR Acupuncture Treatments OR Pharmacoacupuncture Treatment OR Pharmacoacupuncture Therapy OR Acupotomy OR Acupotomies):ab,ti |
| #3 | (Acupuncture, Ear OR Ear Acupunctures OR Auricular Acupuncture OR Auricular Acupunctures):ab,ti |
| #4 | (Electroacupuncture):ab,ti |
| #5 | (Moxibustion OR Moxabustion):ab,ti |
| #6 | #1 OR #2 OR #3 OR #4 OR #5 |
| #7 | (Asthma OR Asthmas OR Bronchial Asthma OR Asthma, Bronchial):ab,ti |
| #8 | #6 AND #7 |

| SEARCH STRATEGY OF WEBN OF SCIENCE | |
| --- | --- |
| #1 | TS=(Acupuncture OR Pharmacopuncture) |
| #2 | TS=(Acupuncture Therapy OR Acupuncture Treatment OR Acupuncture Treatments OR Pharmacoacupuncture Treatment OR Pharmacoacupuncture Therapy OR Acupotomy OR Acupotomies) |
| #3 | TS=(Acupuncture, Ear OR Ear Acupunctures OR Auricular Acupuncture OR Auricular Acupunctures) |
| #4 | TS=(Electroacupuncture) |
| #5 | TS=(Moxibustion OR Moxabustion) |
| #6 | #1 OR #2 OR #3 OR #4 OR #5 |
| #7 | TS=(Asthma OR Asthmas OR Bronchial Asthma OR Asthma, Bronchial) |
| #8 | #6 AND #7 |
